# Supplementary material for: Integral Use of Red Wine Pomace after Hydrostatic High Pressure: Application of Two Consecutive Cycles of Treatment
Source: Foods. 2024 Jan 1;13(1):149. doi: 10.3390/foods13010149 (PMC10778647; doi:10.3390/foods13010149)
Supplement: Supplementary file 1 [file foods-13-00149-s001.zip › foods-2775484-Table S1 delta E color RGP supplementary material.pdf]

Supplementary material

Table S1. Color changes (ΔE) of the high pressure treated RGP stored at different temperatures.

	Refrigeration (4ºC)					Room (20ºC)				
	Control	HHP1	HHP2	2 cycles (a)	2 cycles (b)	Control	HHP1	HHP2	2 cycles (a)	2 cycles (b)
ΔE processing	-	0.3	0.5	0.6	0.8	-	0.3	0.5	0.6	0.8
ΔE storage 1-30d	0.4	0.4	0.3	0.6	0.7	0.5	0.4	0.3	0.7	1.0
ΔE storage 1-90d	0.5	0.5	0.4	0.4	0.6	1.0	1.0	0.8	0.8	0.9
ΔE storage 1-180d	1.0	0.6	0.7	0.4	0.6	2.3	1.4	1.1	1.1	1.2
ΔE storage 1-270d	1.3	1.0	0.5	0.6	0.6	2.0	1.8	1.5	1.4	1.3
